# Limited effects of azithromycin on the oropharyngeal microbiome in children with CF and early pseudomonas infection

**DOI:** 10.1186/s12866-023-03073-8

**Published:** 2023-10-27

**Authors:** Brandie D. Wagner, Edith T. Zemanick, Scott D. Sagel, Charles E. Robertson, Mark J. Stevens, Nicole Mayer-Hamblett, George Retsch-Bogart, Bonnie W. Ramsey, J. Kirk Harris

**Affiliations:** 1 Department of Biostatistics and Informatics, Colorado School of Public Health, University of Colorado, Aurora, CO USA; 2 Children’s Hospital Colorado, Aurora, CO USA; 3 Department of Pediatrics, University of Colorado, Aurora, CO USA; 4 Department of Infectious Diseases, University of Colorado, Aurora, CO USA; 5 Department of Pediatrics, University of Washington, Seattle, WA USA; 6 Department of Biostatistics, University of Washington, Seattle, WA USA; 7 Seattle Children’s Hospital, Seattle, WA USA; 8 Department of Pediatrics, University of North Carolina, Chapel Hill, NC USA

**Keywords:** Microbiome, Oropharyngeal swab, Macrolides, Pulmonary exacerbation, Tobramycin, Beta diversity

## Abstract

**Background:**

Tobramycin inhalation solution (TIS) and chronic azithromycin (AZ) have known clinical benefits for children with CF, likely due to antimicrobial and anti-inflammatory activity. The effects of chronic AZ in combination with TIS on the airway microbiome have not been extensively investigated. Oropharyngeal swab samples were collected in the OPTIMIZE multicenter, randomized, placebo-controlled trial examining the addition of AZ to TIS in 198 children with CF and early *P. aeruginosa* infection. Bacterial small subunit rRNA gene community profiles were determined. The effects of TIS and AZ were assessed on oropharyngeal microbial diversity and composition to uncover whether effects on the bacterial community may be a mechanism of action related to the observed changes in clinical outcomes.

**Results:**

Substantial changes in bacterial communities (total bacterial load, diversity and relative abundance of specific taxa) were observed by week 3 of TIS treatment for both the AZ and placebo groups. On average, these shifts were due to changes in non-traditional CF taxa that were not sustained at the later study visits (weeks 13 and 26). Bacterial community measures did not differ between the AZ and placebo groups.

**Conclusions:**

This study provides further evidence that the mechanism for AZ’s effect on clinical outcomes is not due solely to action on airway microbial composition.

**Supplementary Information:**

The online version contains supplementary material available at 10.1186/s12866-023-03073-8.

## Background

Much of the morbidity and mortality in cystic fibrosis (CF) is due to lung infections, more specifically, p*seudomonas aeruginosa* (PA) is a traditional CF lung pathogen associated with worse clinical outcomes [1]. Consequently, eradication of initial PA infection remains standard of care [2]. Tobramycin inhalation solution (TIS) and chronic Azithromycin (AZ), either alone or in combination, improve clinical outcomes for people with and without chronic PA infection [3–12]. OPTIMIZE (Optimizing Treatment for Early Pseudomonas aeruginosa Infection in Cystic Fibrosis) was a multicenter, randomized, placebo-controlled clinical trial that evaluated whether the addition of AZ to TIS in children with cystic fibrosis and early PA infection decreased the risk of pulmonary exacerbation and prolonged the time to PA recurrence. Prior to the widespread introduction of CFTR modulators, the OPTIMIZE trial showed AZ, in addition to TIS, was associated with a significant reduction in the risk of pulmonary exacerbations (PEx) and an increase in body weight in children with newly acquired PA compared to TIS alone. Despite improved clinical outcomes, there was no difference in rates of PA eradication or recurrence between AZ and placebo treatment groups [4], suggesting the benefits of AZ were not due to improved PA eradication.

Macrolide antibiotics, including AZ, have known anti-inflammatory properties and may have indirect antibacterial activity through alternative mechanisms, such as immunomodulatory, and anti-viral effects, as well as impact on PA biofilm formation, quorum sensing and airway epithelial sodium channel activation, among others [13, 14]. An ancillary study to OPTIMIZE evaluated the effects of randomized AZ treatment on circulating biomarkers of inflammation [15]. A significant decrease in high-sensitivity C-reactive protein was observed at 39 weeks but was not sustained at 78 weeks. Changes in myeloperoxidase, calprotectin and neutrophil count were not different between AZ and placebo groups. Given the demonstrated benefits of AZ without a consistent effect on typical CF pathogens or traditional inflammatory markers to explain the clinical improvement, alternative explanations by which AZ improves pulmonary disease remain of interest.

In the OPTIMIZE study, the addition of AZ to TIS conferred additional clinical benefit, despite no apparent change in PA infection. There is evidence to support that both TIS and AZ may modulate the airway microbiome beyond traditional CF pathogens [16, 17], which may convey clinical benefits outside of their effect on PA. A recent study by Acosta et al. evaluated the microbiome from 38 adults with CF, before and after starting AZ using banked sputum samples [18]. This study found no significant shifts in sputum microbiome related to initiation of AZ suggesting that the observed clinical benefits are unrelated to the modification of the microbial community. These findings were observed in an older CF cohort, where more than half of the study participants started AZ due to concerns of clinical deterioration and other antimicrobial treatments, specifically TIS, could not be tightly controlled. Such limitations necessitate further study to assess the changes of the microbial community with regards to initiation of AZ.

In this ancillary study of oropharyngeal samples collected during the OPTIMIZE clinical trial, we evaluate the impact of TIS with and without AZ on the diversity and composition of the microbiome to determine whether effects on the bacterial community is a potential mechanism of action related to the observed changes in clinical outcomes.

## Results

### Participant and sample characteristics at baseline

**Fig. 1:**
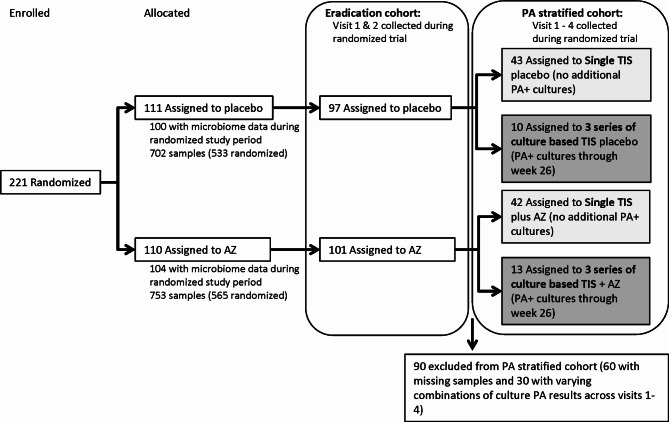
CONSORT diagram. Overview of participants and samples collected for microbiome analysis during the OPTIMIZE trial. Tobramycin inhaled solution (TIS) was utilized based on *Pseudomonas aeruginosa* (PA) culture results. The eradication cohort represents the initial course of TIS with placebo or azithromycin. The PA stratified cohort represents participants that were consistently on or off TIS following the initial eradiation attempt. Visit 1 – baseline, Visit 2 – week 3, Visit 3 – week 13, Visit 4 – week 26, AZ = azithromycin

**Fig. 2:**
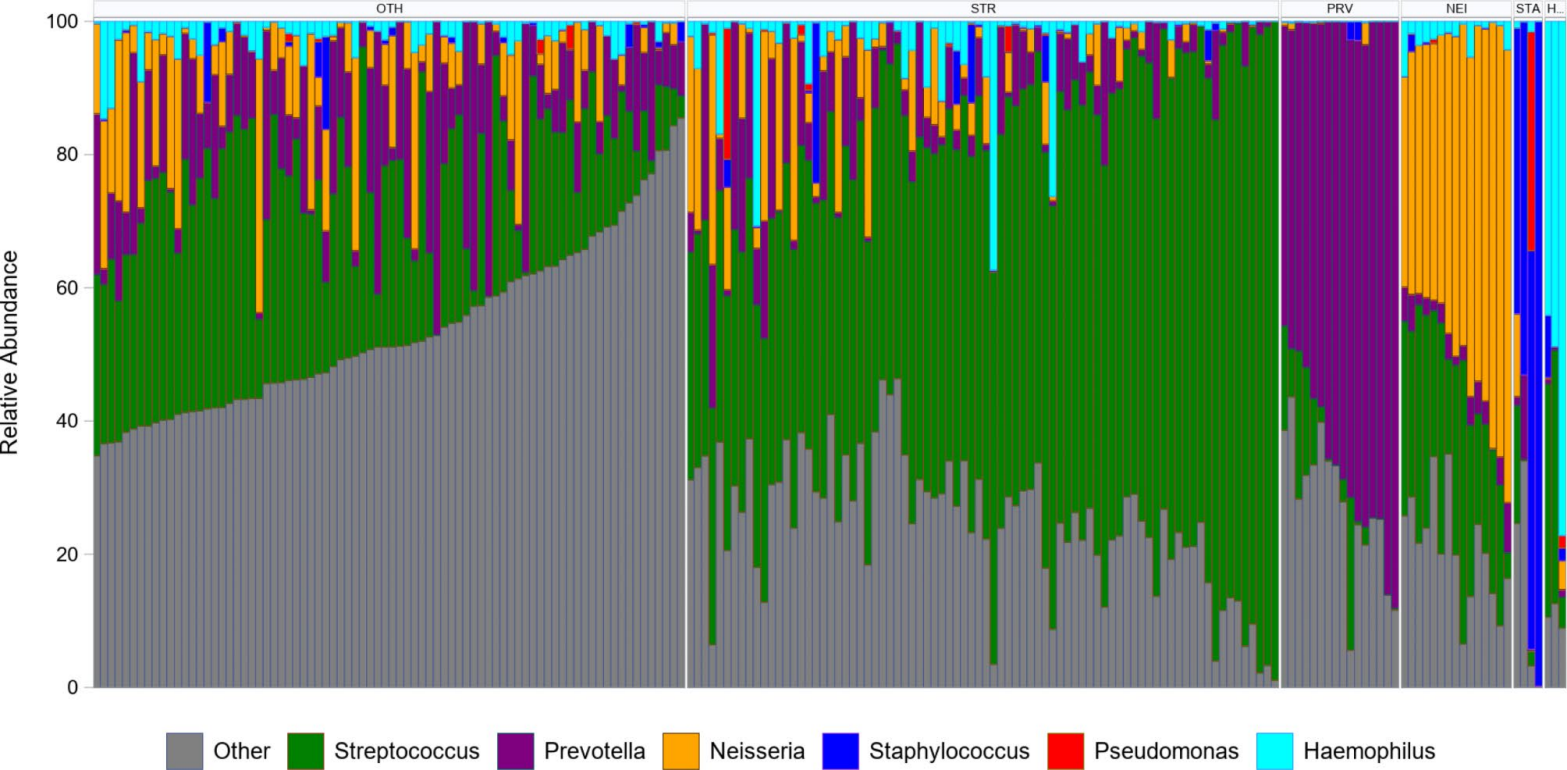
Bacterial composition of baseline samples for all subjects arranged by dominant taxon. *Streptococcus* was the most prominent taxon (n = 80) followed by *Prevotella* (N = 16) and *Neisseria* (n = 15). *Staphylococcus* (n = 4) and *Haemophilus* (n = 3) are shown as representative of CF pathogens. Other is a composite category created by summing all other taxa and was dominant for the 80 samples not included in the selected genera (includes 4 samples each dominated by *Rothia* and *Porphyromonas*). Few samples contain high relative abundance of PA (max RA < 40%). Dominant taxa abbreviations: OTH = Other; STR = Streptococcus; PRV = Prevotella; NEI = Neisseria; STA = Staphylococcus; H = Haemophilus.

Descriptive clinical and demographic information for the eradication and PA stratified cohorts (Fig. 1) are provided in Table 1. At baseline (week 0), PA was detected by culture in 92 participants and by sequencing in 67 participants. PA detection by both methods was observed in 52 participants and PA was not detected by either method in 91 participants. The most abundant genus at baseline was *Streptococcus*, which was the dominant taxa in 80 participants (Fig. 2). In 72 participants, baseline oropharyngeal bacterial communities were not dominated by a single genus. Fewer participants had bacterial communities dominated by other genera including *Prevotella*, *Neisseria*, *Staphylococcus*, *Haemophilus*, *Rothia* and *Porphyromonas*. Oropharyngeal bacterial community composition and ecological metrics at baseline were assessed for differences between analytical groups to confirm randomization did not impact these variables. Neither total bacterial load (TBL) nor Shannon Diversity Index (SDI) differed between treatment groups at the baseline visit for either cohort (Table 2). Sequences indicative of *Streptococcus mitis|oralis|pneumoniae (*herein referred to as the *Streptococcus mitis* group) were the most abundant taxon in OP swabs at baseline across both groups (**Figure **E1).

**Table 1:** Participant demographics by cohorts

	Initial eradication cohort			PA stratified cohort			
N (%) or mean (std)	Azithromycin (N = 101)	Placebo (N = 97)		Single TIS + AZ (N = 42)	Single TIS + Placebo (N = 43)	Three TIS series + AZ (N = 13)	Three TIS series + Placebo (N = 10)
Age in years	6.7 (5)	6.9 (5)		6.1 (5)	7.2 (5)	8.8 (4)	7.5 (6)
Female	52 (51%)	44 (45%)		21 (50%)	21 (49%)	6 (46%)	3 (30%)
Caucasian	92 (91%)	83 (86%)		39 (93%)	38 (88%)	13 (100%)	9 (90%)
Genotype (F508del copy #)							
2	58 (57%)	49 (51%)		28 (67%)	22 (51%)	7 (54%)	5 (50%)
1	28 (28%)	41 (42%)		8 (19%)	19 (44%)	5 (38%)	5 (50%)
0/not done	15 (15%)	7 (7%)		6 (14%)	2 (5%)	1 (8%)	0
FEV_1_% pred	N = 63	N = 56		N = 24	N = 27	N = 11	N = 6
< 75%	5 (8%)	7 (12%)		2 (8%)	2 (8%)	0	1 (17%)
75-100%	31 (49%)	30 (54%)		12 (50%)	16 (59%)	6 (55%)	4 (66%)
>= 100%	27 (43%)	19 (34%)		10 (42%)	9 (33%)	5 (45%)	1 (17%)
First lifetime PA+	60 (59%)	58 (60%)		26 (62%)	22 (51%)	7 (54%)	7 (70%)
CFTR modulator use at baseline (Ivacaftor or Ivacaftor/lumacaftor)	4 (4%)	6 (6%)		1 (2%)	3 (7%)	1 (8%)	1 (10%)
Culture result at baseline*							
* P. aeruginosa*	51 (51%)	41 (44%)		15 (36%)	16 (38%)	11 (85%)	6 (60%)
* S. aureus*	51 (51%)	57 (61%)		22 (52%)	25 (60%)	6 (46%)	5 (50%)
* H. influenzae*	8 (8%)	11 (12%)		4 (10%)	6 (14%)	2 (15%)	0
* S. maltophilia*	2 (2%)	3 (3%)		1 (2%)	2 (5%)	1 (8%)	1 (10%)

*Culture results missing for four subjects in initial eradication cohort and one subject in PA stratified cohort

**Table 2:** Comparison of AZ versus placebo on microbiome outcomes between baseline and week 3

Median (distribution free 95% confidence limits) [p-value^1^]		Azithromycin (N = 101)	Placebo (N = 97)	p-value^2^
TBL	Baseline	6.0 (5.9, 6.1)	6.1 (6.0, 6.3)	0.13
	Change from baseline to week 3	-0.3 (-0.5, -0.2) [p < 0.01]	-0.4 (-0.5, -0.2) [p < 0.01]	0.72
SDI	Baseline	3.3 (3.0, 3.4)	3.5 (3.4, 3.6)	0.12
	Change from baseline to week 3	-0.8 (-1.1, -0.5) [p < 0.01]	-0.6 (-0.9, -0.4) [p < 0.01]	0.16

^1^ p-value assessing whether change from baseline is different from 0 using signed rank test

^2^ p-value testing AZ versus placebo group from Wilcoxon test

TBL = Total Bacterial Load (log_10_ rRNA copy number), SDI = Shannon Diversity Index

### Significant changes in microbial communities occurred at week 3 in both AZ and placebo groups

**Fig. 3:**
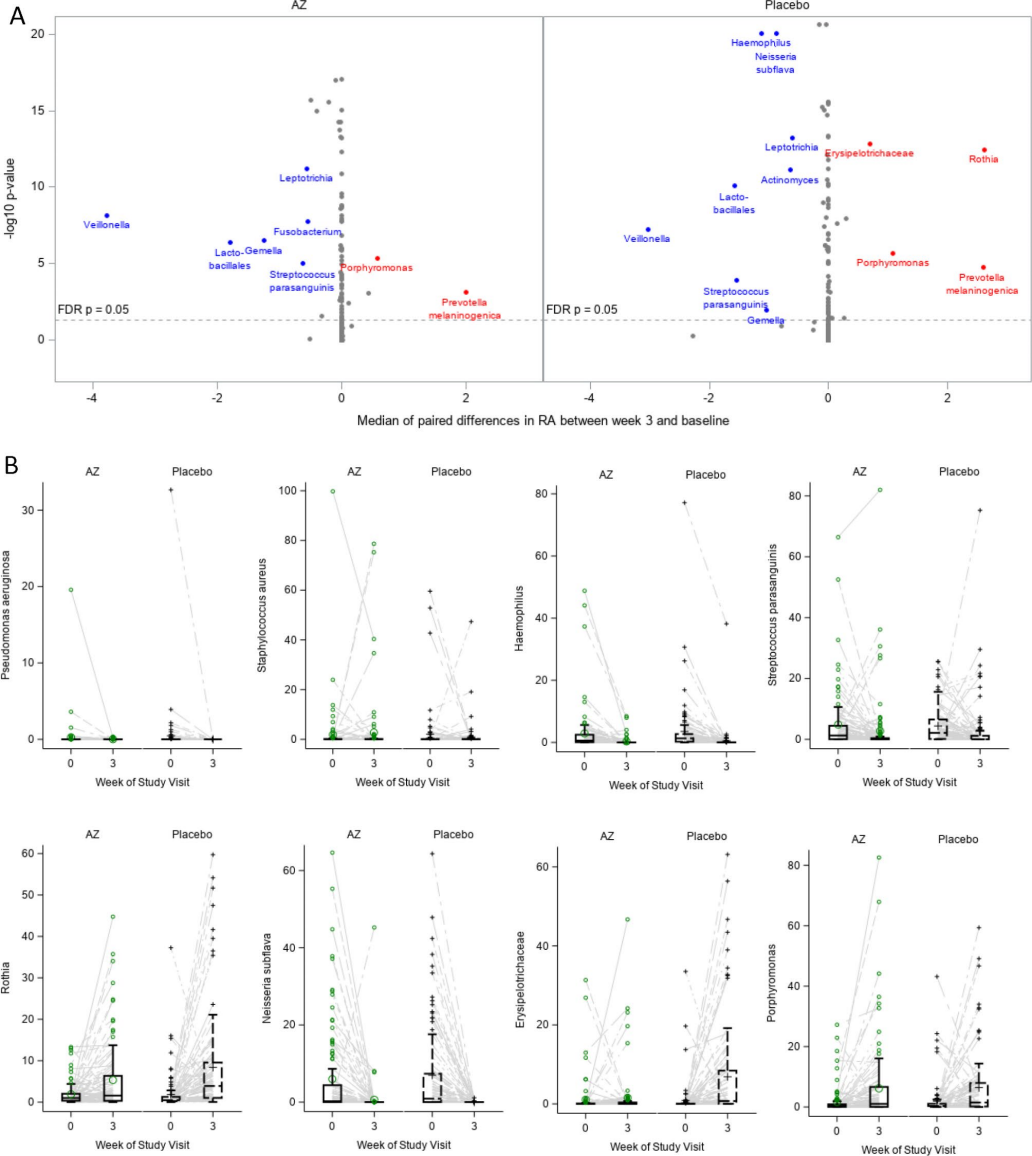
Changes in individual taxa between baseline and week 3. Volcano plot (**A**), points are labeled if FDR p < 0.05 and absolute median estimate greater than 0.5 RA. P-value for volcano plot calculated using signed rank test for each taxa. Changes for individual taxa identified in the volcano plots are displayed (**B**). Placebo + TIS (n = 97); azithromycin + TIS (n = 101) at each visit

Morisita-Horn (MH) and percent of shared taxa (Jaccard) beta diversity were used to compare the bacterial communities between baseline and week 3 samples from the same participant; higher values indicate similarity between the communities. Low beta diversity values indicated significant shifts in the bacterial community in both the community structure and the proportion of shared taxa (Fig. 3). The distribution of beta diversity values did not differ between the AZ and placebo groups. Significant decreases in TBL and SDI after 3 weeks of treatment were observed in both the placebo and AZ groups (**Figure **E2) and these decreases also did not differ by randomized treatment (Table 2). There were no clinically meaningful changes in *Pseudomonas aeruginosa* (PA) or *Staphylococcus aureus* relative abundance (RA) (Fig. 3). Only two participants had notable PA relative abundance at baseline, one in each randomized group, and both relative abundances decreased with treatment. The majority of the participants with high RA for *Staphylococcus aureus* at baseline had a decrease at week 3 but there are instances where this CF pathogen increased. *Haemophilus* also decreased in both AZ and placebo groups. Significant decreases were observed for several other non-traditional CF pathogens including *Veillonella, Lactobacillales*, S*treptococcus parasanguinis*, *Gemella*, *Neisseria subflava* and *Leptotrichia, w*hereas, *Prevotella melaninogenica, Porphyromonas, Rothia* and *Erysipelotrichaceae* increased (**Figure **E3). Changes from baseline to week 3 were similar between the AZ and placebo groups (Fig. 3).

### Changes in microbial communities were not sustained at six month visit

**Fig. 4:**
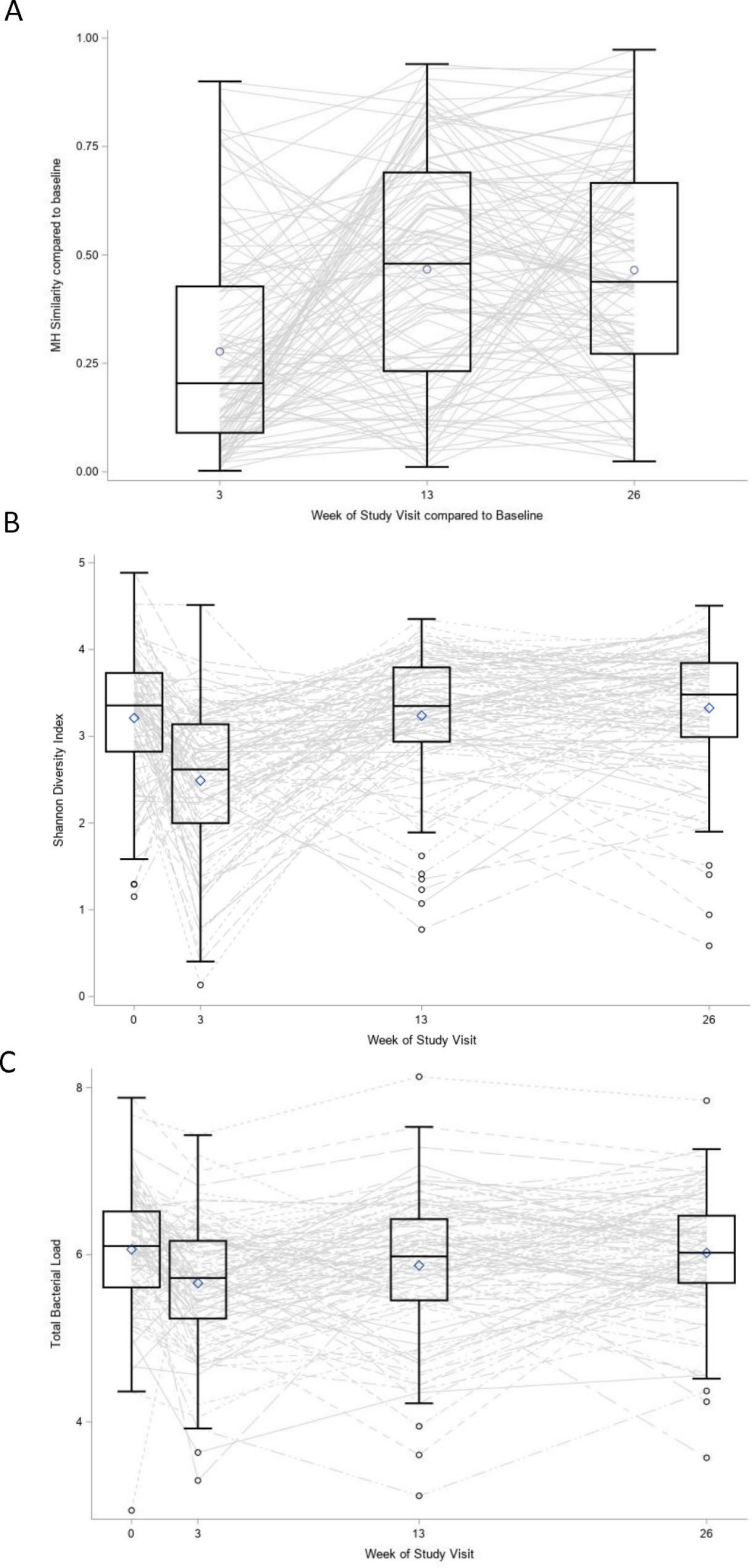
Long term changes in communities were not sustained over 26 weeks. Morisita-Horn (**A**) values compare bacterial compositions between each study visit and baseline. Changes in total bacterial load (**B**) and Shannon Diversity (**C**) observed at week 3 return to baseline levels at the later study visits. Observed values over time are indicated with a line for each individual. Figure includes 108 subjects at each study visit. MH = Morisita-Horn beta diversity

On average, the large shifts in bacterial communities observed at week 3 reverted back to baseline at week 13 and 26. More specifically, MH values comparing later study visits to baseline increased compared to the week 3 comparison. This, in combination with higher MH between weeks 13 and 26, suggests the large shifts in communities at week 3 were not sustained at the later time points (Fig. 4). Similarly, Shannon alpha diversity and total bacterial load returned to baseline at the later study visits. Changes in specific taxa that had large shifts at visit 3 also returned to baseline levels by weeks 13 and 26 (**Figure **E4). These patterns for all microbial measures were present irrespective of TIS or AZ treatment or PA stratification (**Figures **E5, E6, and E7).

### Sustained shifts in microbial communities were observed in a small subset of participants

Given the variability in the bacterial community changes over time, the relevance of these changes was evaluated with regard to treatment differences in clinical outcomes. Changes in bacterial communities at the 3 and 13 week study visits relative to baseline were evaluated using MH (MH_0 − 3_ and MH_0 − 13_). MH values were compared with the PEx outcome, stratified by treatment, to determine the clinical relevance of these shifts. Eight subjects who experienced a PEx prior to week 13 were excluded since their first exacerbation occurred prior to the observed change in their bacterial community, one subject was missing a week 13 sample. After graphical inspection of MH_0 − 13_ versus PEx outcomes, differential effects of treatment appear to occur at low MH values (**Figure **E8). Using a MH cutoff of 0.2 (roughly equal to the 50th percentile for MH_0 − 3_ and the 25th percentile for MH_0 − 13_), proportions of PEx, median number of PEx and time to next PEx are compared across treatments in **Table E3**. For those subjects that had higher MH values (i.e., more stable bacterial communities) there was no difference between treatment groups for any of the PEx outcomes. However, in those with large shifts in their bacterial communities observed at the week 13 study visit, the AZ treatment groups had better PEx outcomes compared to the placebo group, specifically fewer PEx events (42% versus 85%) and longer times to first PEx (**Figure **E9). These effects were present in the subset who also experienced large shifts at the week 3 study visit (**Table E3**). Of the 22 subjects with large shifts in their microbial communities between baseline and 13 weeks MH_0 − 13_ < 0.2), the difference between AZ and placebo groups was not due to a reduction in traditional CF pathogens by graphical inspection (**Figure **E10 & E11).

## Discussion

In this study, the majority of baseline oropharyngeal bacterial communities were either dominated by *Streptococcus* or had high evenness, i.e., not dominated by any single taxa. Despite the eligibility requirement of a PA positive culture, few subjects had detectable PA by sequencing in the baseline visit sample, moreover, the majority also did not have predominance of other CF taxa. Significant decreases in total bacterial load and diversity, as well as substantial changes in bacterial communities were observed by the week 3 study visit for most subjects and did not differ by treatment group (AZ vs. placebo), suggesting these changes are related to TIS. On average, these shifts were due to changes in non-traditional CF taxa that reverted to baseline levels by the 26-week study visit. Decreases in PA and *Staphylococcus aureus* were observed in both treatment groups, but these taxa were only detected in a small number of subjects at baseline. Changes in microbial measures observed at week 3 were not sustained at later visits, both for the cohort with only a single 28-day series of TIS and for the cohort with three culture-based series of 28-day TIS. There was a small subset of subjects (22%) with sustained changes in their bacterial communities at the week 13 study visit that might benefit from AZ. This sub-group may be identifiable by their baseline community which tended to have lower diversity, *Streptococcus* and *Veillonella.* However, these effects do not appear to be directly related to selective pressure on the community composition at the taxonomic level, i.e., the mechanism is not related to the reduction or enrichment of certain taxa.

The OPTIMIZE trial showed a significant reduction in risk of pulmonary exacerbation with inclusion of AZ in addition to TIS for treatment of initial PA infection [4]. Clinical improvements were observed despite no differences in PA eradication rates. Similarly, the TEACH trial also did not observe a decrease in PA density in AZ compared to placebo [19]. The TEACH trial included an older CF age cohort with higher initial PA densities at baseline and evaluated AZ in combination with TIS. These data and results from other studies in CF [5, 11, 18, 20], in addition to the results presented here, provide further evidence that the mechanism for azithromycin’s effect on clinical outcomes is unrelated to antimicrobial action against PA.

Baseline bacterial communities mainly consisted of non-traditional bacteria (anaerobes and bacteria typically classified as oral commensals), despite the enrollment criteria of a positive PA culture. Recent microbiome studies in children have reported similar non-traditional bacteria in cohorts that include sputum from children with HIV [21], in an asthma study that collected hypopharyngeal aspirates [22] and other studies from early PA infection in CF [23]. Sputum from adults with CF collected prior to AZ treatment was more typically dominated by traditional CF pathogens, however a few subjects did exhibit less dominated bacterial communities that included anaerobes, similar to what we observed [18].

Additionally, several randomized clinical trials evaluating the impact of macrolide treatment on oropharyngeal microbial communities in adult cohorts (average age ranged from 38 to 65 across studies) outside of CF have been reported [24–27]. Similar to our findings, the baseline communities in all of these studies were dominated by *Streptococcus* and *Prevotella*. A change in total bacterial load was not reported after 48 weeks of a macrolide in a study of non-CF bronchiectasis [25]. Changes in alpha diversity measures are discrepant between studies, one found that evenness decreased [25], whereas no changes were observed in a cohort of healthy individuals [26]. Similarly, the results of studies differ with regards to changes in individual taxa, specifically *Streptococcus* and *Haemophilus*, some indicated significant changes in these taxa [25–27] whereas others did not [24]. More generally, the resilience of the airway microbiome associated with antibiotic related perturbations has been observed in other CF studies, including both pediatric and adult cohorts [28–31].

Similar to our finding, a recent study using banked sputum samples [18] did not observe major impacts on the bacterial communities following 2 years of AZ treatment. However, decreases in *Prevotella* and increases in *Haemophilus* were reported. This is in contrast to our study that showed an increase in *Prevotella* and a decrease in *Haemophilus* at the week 3 study visit then a return to baseline levels. These discrepancies may be due to the longer follow-up time of the Acosta et al. study, or the reason for initiation of AZ treatment, as more than half of the patients started AZ related to concerns of clinical deterioration. Additionally, TIS was more strictly controlled in OPTIMIZE, whereas this wasn’t possible in the Acosta study. Our findings suggest that the large changes in the microbiome may be attributed to TIS rather than to AZ. Previous studies evaluating microbiome changes due to TIS similarly found shifts in bacterial communities that were not sustained over time [16, 17]. Furthermore, shifts in taxonomy were not correlated with functional differences by metagenomics [17].

While the prevalence of lung infections decreases with highly effective CFTR modulators [32], prospective studies suggest that infection persists after initiation of ivacaftor [33, 34] or elexacaftor/tezacaftor/ivacaftor [35, 36]. Evidence for changes in airway inflammation with highly effective modulators is mixed and prospective studies are ongoing [37]. Thus, antimicrobial and anti-inflammatory therapies, including AZ, are likely to remain a valuable tool for managing CF lung disease and understanding the mechanism of activity may influence use in modulator treated populations.

Major strengths of this study include the large multicenter cohort of young children with CF enrolled soon after initial PA infection, longitudinal study design with multiple collected oropharyngeal samples, consistent administration of concurrent TIS and was ancillary to a randomized clinical trial that allows a more direct inference of causal relationships. However, it is not without limitations, OP samples were used to describe airway bacterial communities and may not be representative of the lower airway in all participants [38]. Despite this, OP samples remain used clinically to manage PA airway infection in children with CF and are more feasible than collecting lower airway samples. Further, our results aligned with previous studies of AZ using sputum samples collected from older CF individuals [18], which partially addresses this limitation. Our study is unable to make any inferences regarding potential functional or metabolic shifts in the bacterial communities or impacts on other components of the microbiome (including viruses or fungi) as only total bacterial load and microbial taxonomic composition were assessed. Some of the comparisons were based on small sample sizes and require further validation.

## Conclusion

Our findings suggest that the impact on clinical outcomes observed during the OPTIMIZE trial was not due to antimicrobial effect by AZ on the oropharyngeal bacterial community composition even in a younger cohort randomized to treatment and accounting for concurrent TIS use. The mechanism of action for AZ is unlikely to be directly affecting the airway microbiome.

## Methods

An expanded Methods section is available in the online supplement.

### Study design

The parent OPTIMIZE study was a multicenter, randomized, double-blind, placebo-controlled clinical trial in children with CF who were 6 months to 18 years of age at the time of new isolation of PA from respiratory-tract cultures (OPTIMIZE NCT02054156) [4]. Study participants with a new positive PA culture were treated with a 28-day course of TIS and an additional 28 days of TIS treatment for those who remained PA positive at 21 days. Over the remaining quarters of the study, treatment with TIS was given for an additional 28 days only when participants tested positive for PA. Study participants were randomized to receive either azithromycin three times weekly or matched placebo over the entire 18-month study period.

The OPTIMIZE trial included 221 randomized subjects. For this ancillary study, data from participants with missing baseline oropharyngeal (OP) samples or with fewer than 2 OP samples were excluded, resulting in 198 participants. OP samples were obtained from all participants up to eight visits over the 78-week study (n = 1,457 swabs). The OPTIMIZE trial was converted to an open label observational study after demonstrating significantly decreased rates of PEx in the AZ group as compared to placebo during an interim analysis. The analysis described here was limited to the subset of swabs collected during the randomized-blinded portion of OPTIMIZE (Fig. 1: CONSORT diagram). Two analytic cohorts were generated using a subset of data from the randomized portion of the trial. The first cohort included 198 participants comprised of those with samples collected at both baseline and 3 weeks after randomization (initial eradication analytic cohort). All subjects in the initial eradication cohort were concurrently on TIS, thus allowing effects of AZ to be assessed in combination with TIS. The second cohort examined the impact of azithromycin over 26 weeks. Per the study protocol, participants with PA positive cultures at any visit after week 3 were provided a 28-day course of TIS at that visit. The second analytic cohort was generated after stratifying participants by exposure to culture based TIS (stratified PA analytic cohort) and consisted of a group that only received a single TIS treatment and a group that received 3 series of culture based TIS. This second analytic cohort contained 432 samples from 108 participants to evaluate the impact of azithromycin over 6-months, holding TIS exposure constant (Fig. 1). Data for the swabs collected during the observational phase are included in the online supplement. The swabs were frozen at -80 °C and batch shipped on dry ice to the Cystic Fibrosis Microbiome Analysis Core, a CF Foundation funded national resource center at Children’s Hospital Colorado and University of Colorado Anschutz Medical Campus for microbiome analysis.

### Microbiome

DNA was extracted after enzymatic digestion [39, 40] using the EZ1 Advanced extraction platform (Qiagen). Total bacterial load was determined by the Nadkarni qPCR assay [41, 42]. Bacterial community profiles were determined by sequence analysis of V1/V2 16S rRNA gene fragment following our previously described methods [1, 43]. Species assignments for specific genera (*Pseudomonas, Staphylococcus, Prevotella, Neisseria and Streptococcus*) were made based on high levels of sequence identity (> 99.9%) using heavily curated reference sequences [44]. In cases where multiple species are indistinguishable by 16S rRNA gene sequence, groups of species were assigned. Sequences not affiliated with a species remain assigned at the genus level. Community profiles were used to calculate alpha diversity metrics (Shannon Diversity Index, Shannon Evenness) and relative abundance of community constituents. Community profiles were compared using the Morisita-Horn beta diversity metric.

### Statistical analyses

Comparisons in microbiome outcomes (total bacterial load (TBL), Shannon Diversity Index (SDI), Morisita-Horn beta diversity (MH) and the relative abundances of individual taxa) across treatment groups were evaluated using non-parametric rank-based asymptotic tests with continuity corrections (Wilcoxon and Kruskal-Wallis tests and signed rank tests for within subject changes over time). Medians and distribution free 95% confidence limits are presented for microbial measures. In addition, exploratory data analysis that included graphical inspection and principal coordinates analysis (PCOA) was used to assess correlation with clinical factors. The Morisita-Horn and Jaccard beta diversity measures were compared across groups using a beta regression model. Kaplan-Meier curves were used to evaluate time to first pulmonary exacerbation. All analyses were performed using SAS version 9.4 (The SAS Institute, Cary, NC) and R version 3.4.1 (The R Foundation for Statistical Computing, Vienna, Austria).

### Electronic supplementary material

Below is the link to the electronic supplementary material.

**Table E1**. Changes in microbiome measures from baseline in the PA stratified cohort. **Table E2**. Describe low MH cohort. **Table E3**. Effect of AZ treatment on PEx outcomes stratified by changes in microbial composition. **Figure E1**. Compare bacterial communities between randomized groups at baseline. Heatmap with taxa presented in rows, samples in columns, the darker blue indicates higher RA. **Figure E2**. Initial eradication cohort. Large shifts in the communities between week 0 and 3 were observed. Morisita-Horn (A) values compare bacterial compositions between week 3 visit and baseline, MH values typically observed between replicates is indicated by the shaded area. Changes in the proportion of shared taxa between paired samples (B) averaged around 45%. Although these changes are significantly different across treatment groups the magnitude of difference is not clinically meaningful. Decreases were observed in both total bacterial load (C) and Shannon Diversity (D) after three weeks of treatment with either Placebo +TIS (n = 97) or azithromycin +TIS (n = 101). AZ=azithromycin; MH=Morisita-Horn beta diversity. **Figure E3**. Distributions of differences between week 3 and baseline. Boxplots of the differences in RA for select taxa by treatment group are displayed. AZ=azithromycin. **Figure E4**. Bacterial communities over time. Heatmap with taxa presented in rows, samples in columns, the darker blue indicates higher RA. **Figure E5**. Distribution of MH by PA stratified cohorts comparing communities at each study visit to baseline. **Figure E6**. Distribution of change in TBL and SDI over time by PA stratified cohorts. TBL=total bacterial load; SDI = Shannon Diversity Index. **Figure E7**. Change from baseline for microbial measures by PA stratified cohorts. **Figure E8**. PEx outcomes (any PEx (top) 0=no, 1=yes; number of PEx (bottom)) plotted versus MH0-13 stratified by treatment. **Figure E9**. Time to PEx based on initial eradication treatment group stratified by community stability (MH < 0.2). bacterial community profiles for 22 individuals with large shifts in community composition between baseline and week 13 had differential response to treatment. **Figure E10**. Microbial community composition for 22 subjects with low MH0-13. Each subject is displayed in a separate panels showings amples from each study visit. Labels on top of panels represent the randomized treatment group, age, de-identified id, number and days to 1st PEx (or censoring time if 0 PEx). **Figure E11**. PCoA showing whether the 22 subjects with low MH0-13 are converging to a similar community (top), subjects with higher MH0-13 are plotted for comparison (bottom).

## Data Availability

The clinical data that support the findings of this study are available from the Cystic Fibrosis Foundation (CFF) but restrictions apply to the availability of these data, which were used under license for the current study, and so are not publicly available. Data are however available from the authors upon reasonable request and with permission of CFF. The DNA sequencing data reported in this paper have been deposited in the NCBI Short Read Archive database under accession number PRJNA954039 following guidelines from the National Center for Biotechnology Information.

## Abbreviations

AZ: Azithromycin
CF: Cystic Fibrosis
MH: Morisita-Horn
OP: Oropharyngeal
PA: Pseudomonas aeruginosa
PCOA: Principal coordinates analysis
PEx: Pulmonary exacerbation
RA: Relative abundance
SDI: Shannon diversity index
TBL: total bacterial load
TIS: Tobramycin inhalation solution
