# Supplementary material for: Limited effects of azithromycin on the oropharyngeal microbiome in children with CF and early pseudomonas infection
Source: BMC Microbiol. 2023 Oct 27;23:312. doi: 10.1186/s12866-023-03073-8 (PMC10612347; doi:10.1186/s12866-023-03073-8)
Supplement: Supplementary file 1 — Table E1. Changes in microbiome measures from baseline in the PA stratified cohort. Table E2. Describe low MH cohort. Table E3. Effect of AZ treatment on PEx outcomes stratified by changes in microbial composition. Figure E1. Compare bacterial communities between randomized groups at baseline. Heatmap with taxa presented in rows, samples in columns, the darker blue indicates higher RA. Figure E2. Initial eradication cohort. Large shifts in the communities between week 0 and 3 were observed. Morisita-Horn (A) values compare bacterial compositions between week 3 visit and baseline, MH values typically observed between replicates is indicated by the shaded area. Changes in the proportion of shared taxa between paired samples (B) averaged around 45%. Although these changes are significantly different across treatment groups the magnitude of difference is not clinically meaningful. Decreases were observed in both total bacterial load (C) and Shannon Diversity (D) after three weeks of treatment with either Placebo +TIS (n = 97) or azithromycin +TIS (n = 101). AZ=azithromycin; MH=Morisita-Horn beta diversity. Figure E3. Distributions of differences between week 3 and baseline. Boxplots of the differences in RA for select taxa by treatment group are displayed. AZ=azithromycin. Figure E4. Bacterial communities over time. Heatmap with taxa presented in rows, samples in columns, the darker blue indicates higher RA. Figure E5. Distribution of MH by PA stratified cohorts comparing communities at each study visit to baseline. Figure E6. Distribution of change in TBL and SDI over time by PA stratified cohorts. TBL=total bacterial load; SDI = Shannon Diversity Index. Figure E7. Change from baseline for microbial measures by PA stratified cohorts. Figure E8. PEx outcomes (any PEx (top) 0=no, 1=yes; number of PEx (bottom)) plotted versus MH0-13 stratified by treatment. Figure E9. Time to PEx based on initial eradication treatment group stratified by community stability (MH < 0.2). ba [file 12866_2023_3073_MOESM1_ESM.docx]

**IMPACT OF AZITHROMYCIN ON THE AIRWAY MICROBIOME IN CHILDREN WITH CF AND EARLY PSEUDOMONAS INFECTION**

**ONLINE DATA SUPPLEMENT**

**METHODS**

**Sample collection and storage:** A research oropharyngeal (OP) swab was collected in parallel with clinical surveillance swabs for bacterial culture obtained at study visits. The clinical sample was sent to the local clinical microbiology laboratory for standard CF microbiologic culture (2); the research swab was frozen at -70°C or below and then batch shipped on dry ice to the Cystic Fibrosis Microbiome Analysis Laboratory at Children’s Hospital Colorado for molecular analyses. Swabs were stored at -80°C until processed. Swabs were transferred to 4°C to thaw prior to processing.

**Swab processing:** Each swab was cut into a sterile 2ml tube containing 0.39 mL of phosphate buffered saline and vortexed on high for 1 min. Next, 0.05 mL each of lysozyme (174,000 units) and lysostaphin (105 units) were added, and the mixture incubated at 37°C for 30 min. Proteinase K digestion was performed at 65°C using 0.01 mL solution provided with the extraction kit (Qiagen). Enzymes were inactivated using a 10 min 95°C incubation step, and then the samples were cooled to 4°C to facilitate handling. An aliquot for extraction (0.2 mL) was prepared and frozen at -20°C until extraction was performed. The remaining aliquot was stored at -80°C. All reagents used for enzymatic digestion were first extracted and assayed by TBL qPCR (below) to assess bacterial DNA present in the reagents. The bacterial load was indistinguishable from the extraction kit for all reagents utilized.

**DNA Extraction:** DNA extraction was performed using the Qiagen EZ1 Advanced platform. The Bacterial DNA card and Tissue Extraction kit were used per manufacturer’s instructions. DNA was eluted in 0.1 mL of elution buffer and stored at -20°C during subsequent amplification steps (<2 weeks). Long term storage was at -80°C. Extraction controls were performed using water (reagent used for PCR) and extraction buffer (G2; not utilized in this study). Each lot of the Qiagen kit was pre-certified before use with clinical samples.

**Total Bacterial Load:** We utilized the qPCR described by Nadkarni and previously evaluated in CF airway samples to estimate the amount of bacterial DNA present in each sample (3, 4). This assay uses TaqMAN probe (~eub338) approach with amplification of ~500 nt (~27F/~515R) of the small subunit rRNA gene. DNA extracts were diluted 1:40, and 4 µL of template (dilution factor of 10) were assayed in triplicate for each extraction. The standard curve was generated using a cloned 16S rRNA gene with copy numbers from 10^3^ to 10^8^ copies per reaction. The background was assessed using blanks assayed in triplicate on each plate of qPCR; the average copies per reaction 49.33 (1.7 log_10_; standard deviation 48.67), and the maximum background measurement was 330 copies per reaction (2.5 log_10_).

**Amplicon Generation:** Each DNA extract was amplified in triplicate along with an index specific negative PCR (single mastermix). Each reaction contained 1X HotmasterMix (5Prime), 150 nM each indexed 27F and 338R primer in a 25 µL reaction volume. Cycling conditions were 94°C 2 min followed by 30 cycles of 94°C 20 sec, 52°C 20 sec, 65°C 60 sec. After thermal cycling the amplicons were assessed by agarose gel electrophoresis (pooled triplicates and negative control) for appropriate sized bands from the DNA template and no evidence of amplification from the negative control. If any amplification was evident in the negative control the assay for that DNA extract was repeated. Both PCR and extraction controls were included in sequencing pools. There was no apparent amplification from either type of controls based on agarose gel electrophoresis.

**Sequencing:** PCR products were normalized using agarose gel densitometry. An approximately equimolar amount of each sample was pooled into a single mixture (controls were below detection and added at maximal volume, 20 µL). The pool was gel purified using 1.5% agarose gel (Tris Acetate EDTA, TAE) per the manufacturer’s instructions (Montage). The gel purified pool was concentrated using a DNA Clean and Concentrator Kit (Zymo, Irvine, CA), which also exchanges electrophoresis buffer with Tris-EDTA. Pooled amplicons were quantified using Qubit Fluorometer 2.0 (Invitrogen, Carlsbad, CA). The pool was diluted to 4nM and denatured with fresh 0.2 N NaOH at room temperature. The denatured DNA was diluted to 20pM and spiked with 10% PhiX control DNA (Illumina) prior to loading the sequencer. Illumina paired-end sequencing was performed on the MiSeq platform using a 500-cycle version 2 reagent kit (Illumina).

**Sequence analysis:** Illumina MiSeq paired-end reads were aligned to human reference genome Hg19 with bowtie2 and matching sequences discarded (5, 6). (5, 6). As previously described (7-9), the remaining non-human paired-end sequences were sorted by sample via barcodes in the paired reads with a python script (8). Sorted paired end sequence data were deposited in the NCBI Short Read Archive under bioproject accession number PRJNA954039. The sorted paired reads were assembled using phrap (10, 11). Pairs that did not assemble were discarded. Assembled sequence ends were trimmed over a moving window of 5 nucleotides until average quality met or exceeded 20. Trimmed sequences with any ambiguity or shorter than 200 nt were discarded. Potential chimeras identified with Uchime (usearch6.0.203_i86linux32) (12) using the Schloss (13) Silva reference sequences were removed from subsequent analyses. Assembled sequences were aligned and classified with SINA (1.3.0-r23838) (14) using the 418,497 bacterial sequences in Silva 115NR99 (15) as reference configured to yield the Silva taxonomy. Sequences with identical taxonomic assignments were grouped to produce Operational taxonomic units (OTUs). This process generated 153,967,184 sequences for 1,052 samples (average sequence length: 316 nt; average sample size: 146,218 sequences/sample; minimum sample size: 66,348; maximum sample size: 401,643). The median Goods coverage score was ≥ 99.95% at the rarefaction point of 66,348. The software package Explicet (v2.10.5, www.explicet.org) (16) was used for data visualization, analysis (rarefied values for median Good’s coverage, Shannon diversity index, Morisita-Horn Beta diversity), and select figure generation.

**RESULTS**

Comparison of PA culture versus sequencing

Pseudomonas aeruginosa (PA) culture results were available from 1,091 visits (1,002 cultures from OP, 88 from sputum, 1 BAL). PA was detected by sequencing in 68 (8%) samples that were culture negative; and was not detected by sequencing in 145 (52%) samples that were culture positive. Detection by both methods occurred in 136 (12%) samples. The relative abundance in those that were detected by sequencing alone was low (median, range: 0.003 (0.001 – 0.34) compared to the samples where the methods agreed (0.01 (0.001 - 80.13).

**Table E1**. Changes in microbiome measures from baseline in the PA stratified cohort

|  | | Single 28-day TIS | | Multiple TIS (3 series of 28 day treatments) | |  |
| --- | --- | --- | --- | --- | --- | --- |
| Median (distribution free 95% confidence limits) [p-value^1^] | | Azithromycin  (N = 42) | Placebo  (N = 43) | Azithromycin  (N = 13) | Placebo  (N = 10) | p-value^2^ |
| TBL (log_10_ rRNA copy number) | Baseline | 6.0 (5.8, 6.4) | 6.1 (6.0, 6.4) | 6.2 (5.5, 6.4) | 6.0 (5.3, 6.9) | 0.90 |
|  | Change from baseline to week 3 | -0.3 (-0.7, -0.1)  [p=0.02] | -0.4 (-0.7, -0.2) [p<0.01] | -0.4 (-0.9, -0.2) [p<0.01] | 0 (-1.2, 0.7) [p=0.70] | 0.69 |
|  | Change from baseline to week 13 | 0 (-0.6, 0.2) [p=0.33] | -0.1 (-0.2, 0.1) [p=0.29] | -0.6 (-0.8, 0) [p=0.03] | -0.2 (-1.0, 1.1) [p=0.85] | 0.58 |
|  | Change from baseline to week 26 | -0.2 (-0.5, 0.4) [p=0.94] | 0.1 (-0.3, 0.3) [p=0.98] | 0 (-0.6, 0.2) [p=0.74] | 0.2 (-1.2, 0.5) [p=0.70] | 0.97 |
|  |  |  |  |  |  |  |
| SDI | Baseline | 3.3 (3.0, 3.4) | 3.4 (3.3, 3.7) | 3.0 (2.4, 3.7) | 3.5 (2.4, 3.9) | 0.36 |
|  | Change from baseline to week 3 | -1.0 (-1.5, -0.3)  [p <0.01] | -0.6 (-0.9, -0.1) [p<0.01] | -1.0 (-1.5, 0.1) [p=0.02] | -0.7 (-1.9, -0.4) [p=0.03] | 0.39 |
|  | Change from baseline to week 13 | 0.2 (-0.2, 0.5) [p=0.20] | -0.1 (-0.3, 0.3) [p=0.54] | 0.1 (-0.9, 0.9) [p=0.99] | 0.2 (-0.8, 0.9) [p=0.56] | 0.57 |
|  | Change from baseline to week 26 | 0.2 (0, 0.5) [p=0.08] | 0.1 (-0.2, 0.4) [p=0.53] | -0.2 (-0.8, 1.0) [p=0.79] | 0 (-1.0, 0.7) [p=0.85] | 0.69 |

^1^ p-value assessing whether change from baseline is different from 0 using signed rank test

^2^ p-value testing AZ versus placebo group from Wilcoxon test

**Table E2. Describe low MH cohort**

|  | MH0-13 < 0.2  (N = 22) | MH0-13 > 0.2  (N = 77) | p-value |
| --- | --- | --- | --- |
| Randomized to AZ | 11 (50%) | 39 (51%) | 0.96 |
| Age in years, mean (std) | 5.7 (4.3) | 7.6 (5.0) | 0.13 |
| Sex=2, female | 7 (32%) | 38 (49%) | 0.22 |
| Caucasian | 20 (91%) | 73 (95%) | 0.61 |
| Genotype | | | 0.81 |
| F508 homo | 14 (64%) | 43 (56%) |  |
| F508 hetero | 7 (32%) | 27 (35%) |  |
| Other/not done | 1 (4%) | 7 (9%) |  |
| Baseline FEV% pred | | | 0.44* |
| < 75% | 2 (15%) | 3 (6%) |  |
| 75%-100% | 7 (54%) | 30 (58%) |  |
| >= 100% | 4 (31%) | 19 (37%) |  |
| Culture | | | |
| Pa | 10 (45%) | 35 (46%) | 0.96 |
| SA | 13 (59%) | 37 (49% | 0.39 |
| Hflu | 4 (18%) | 8 (11%) | 0.46* |
| SM | 0 | 5 (7%) | 0.58* |
| Microbial markers | | | |
| TBL, mean (std) | 6.0 (0.5) | 6.1 (0.8) | 0.81 |
| SDI, mean (std) | 2.8 (0.8) | 3.3 (0.7) | **<0.01** |
| Richness, med [IQR] | 45 [39-66] | 56 [47-66] | 0.29 |
| Veillonella, med [IQR] | 0.77 [0.05-4.54] | 6.99 [3.62 – 16.83] | **<0.01** |
| Streptococcus, med [IQR] | 2.25 [0.23-7.29] | 7.02 [3.92-13.23] | **<0.01** |
| Prevotella, med [IQR] | 0.57 [0.26-1.29] | 0.37 [0.16-0.78] | 0.20 |
| Haemophilus, med [IQR] | 0.11 [0.06-0.14] | 1.15 [0.17-2.70] | 0.09 |

-excludes 8 subjects with PEX prior to 13 week study visit; 1 subject is missing a week 13 sample

*Fisher’s exact test was used to calculate p-value

**Table E3. Effect of AZ treatment on PEx outcomes stratified by changes in microbial composition**

|  | | MH <=0.2 | | | MH >0.2 | | |
| --- | --- | --- | --- | --- | --- | --- | --- |
|  |  | Azithromycin | Placebo | p-value | Azithromycin | Placebo | p-value |
| MH0-3 | PE | 13 (45%) | 15 (60%) | 0.29 | 12 (46%) | 17 (61%) | 0.28 |
|  | PE count | 0 [0-1] | 1 [0-1] | 0.44 | 0 [0-2] | 1 [0-1] | 0.70 |
|  | Time to PE | 324 [138-493] | 285 [204-371] | 0.70 | 367 [258-531] | 249 [178-401] | 0.08 |
| MH0-13 | PE | 5 (42%) | 11 (85%) | **0.04** | 20 (47%) | 21 (53%) | 0.59 |
|  | PE count | 0 [0-1] | 1 [1-2] | **0.03** | 0 [0-1] | 1 [0-1] | 0.79 |
|  | Time to PE | 464 [263-546] | 187 [99-240] | **0.01** | 301 [150-505] | 315 [200-423] | 0.98 |
| MH0-13\|0-3 <0.2 | PE | 2 (33%) | 6 (75%) | 0.28 | 10 (45%) | 9 (53%) | 0.64 |
|  | PE count | 0 [0-1] | 1 [1-5] | 0.13 | 0 [0-1] | 1 [0-1] | 0.91 |
|  | Time to PE | 430 [202-555] | 156 [62-258] | 0.14 | 270 [119-476] | 347 [281-418] | 0.36 |


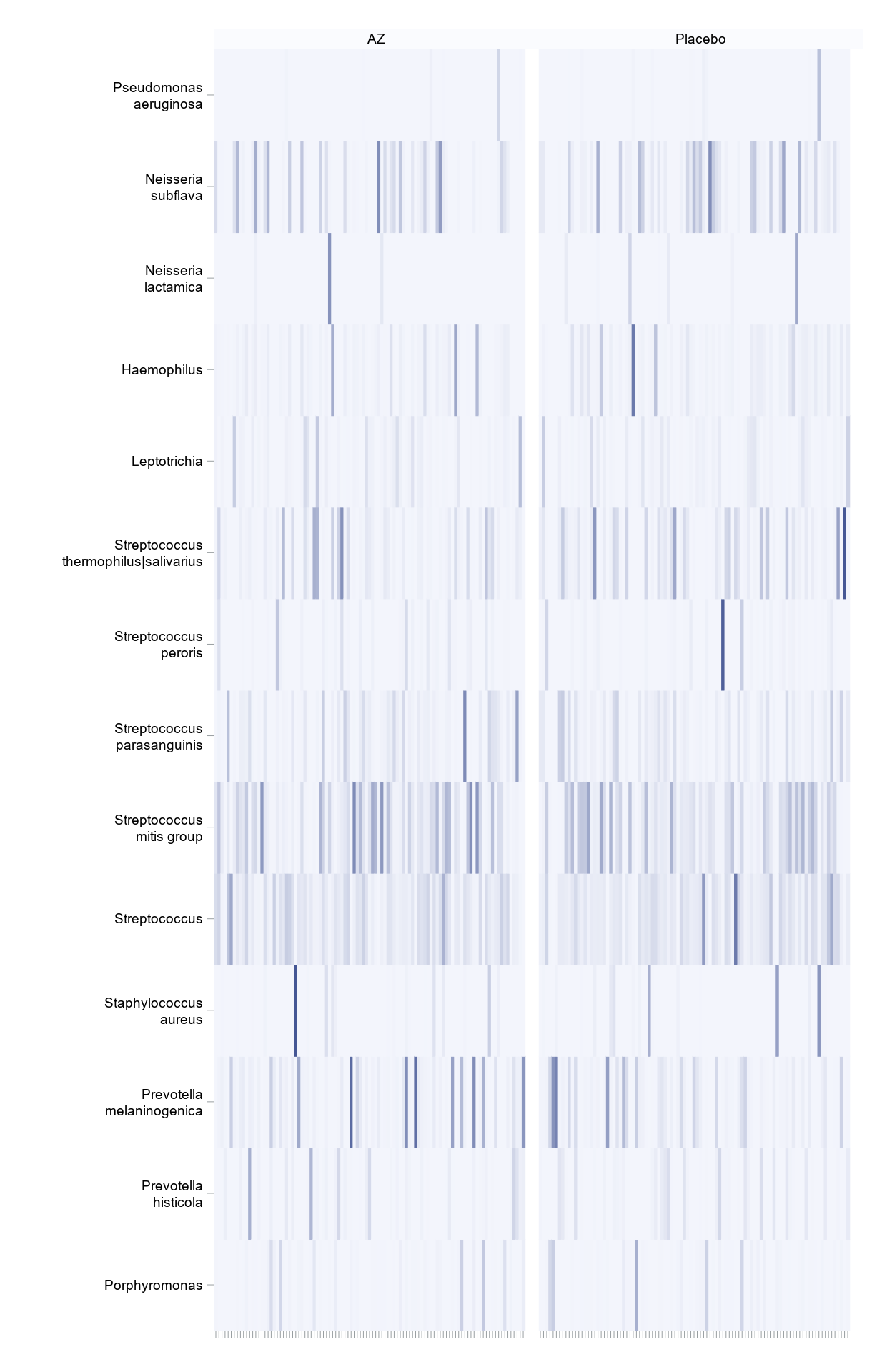


Figure E1. Compare bacterial communities between randomized groups at baseline. Heatmap with taxa presented in rows, samples in columns, the darker blue indicates higher RA.


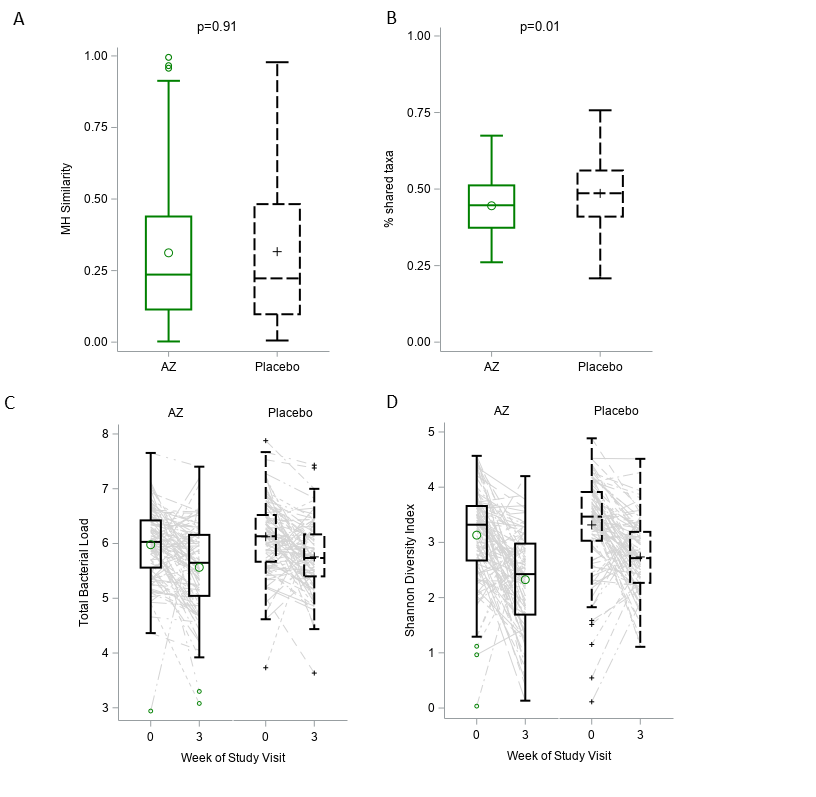
Figure E2. Initial eradication cohort. Large shifts in the communities between week 0 and 3 were observed. Morisita-Horn (A) values compare bacterial compositions between week 3 visit and baseline, MH values typically observed between replicates is indicated by the shaded area. Changes in the proportion of shared taxa between paired samples (B) averaged around 45%. Although these changes are significantly different across treatment groups the magnitude of difference is not clinically meaningful. Decreases were observed in both total bacterial load (C) and Shannon Diversity (D) after three weeks of treatment with either Placebo +TIS (n = 97) or azithromycin +TIS (n = 101). AZ=azithromycin; MH=Morisita-Horn beta diversity


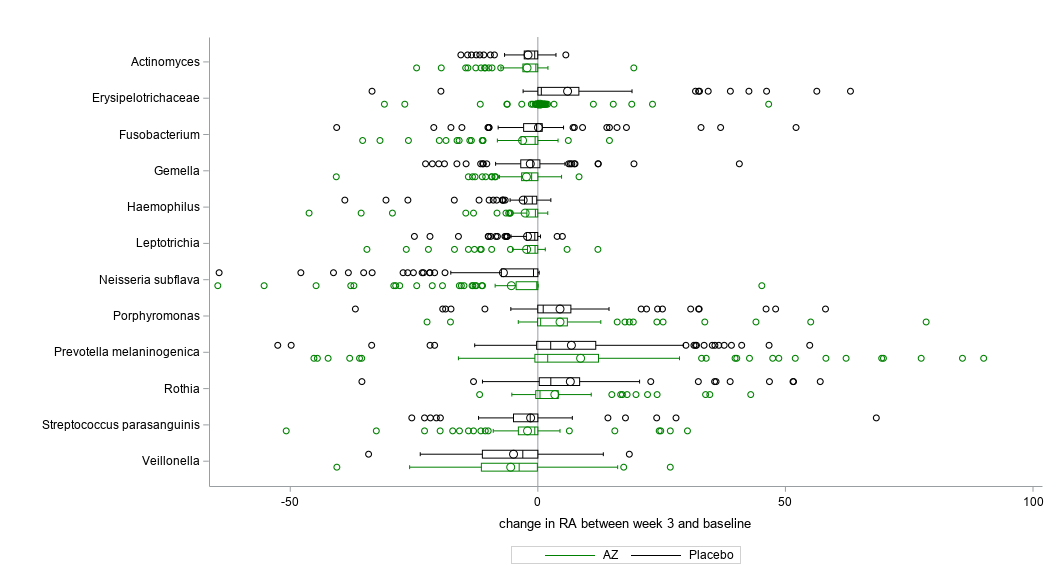


Figure E3. Distributions of differences between week 3 and baseline. Boxplots of the differences in RA for select taxa by treatment group are displayed. AZ=azithromycin


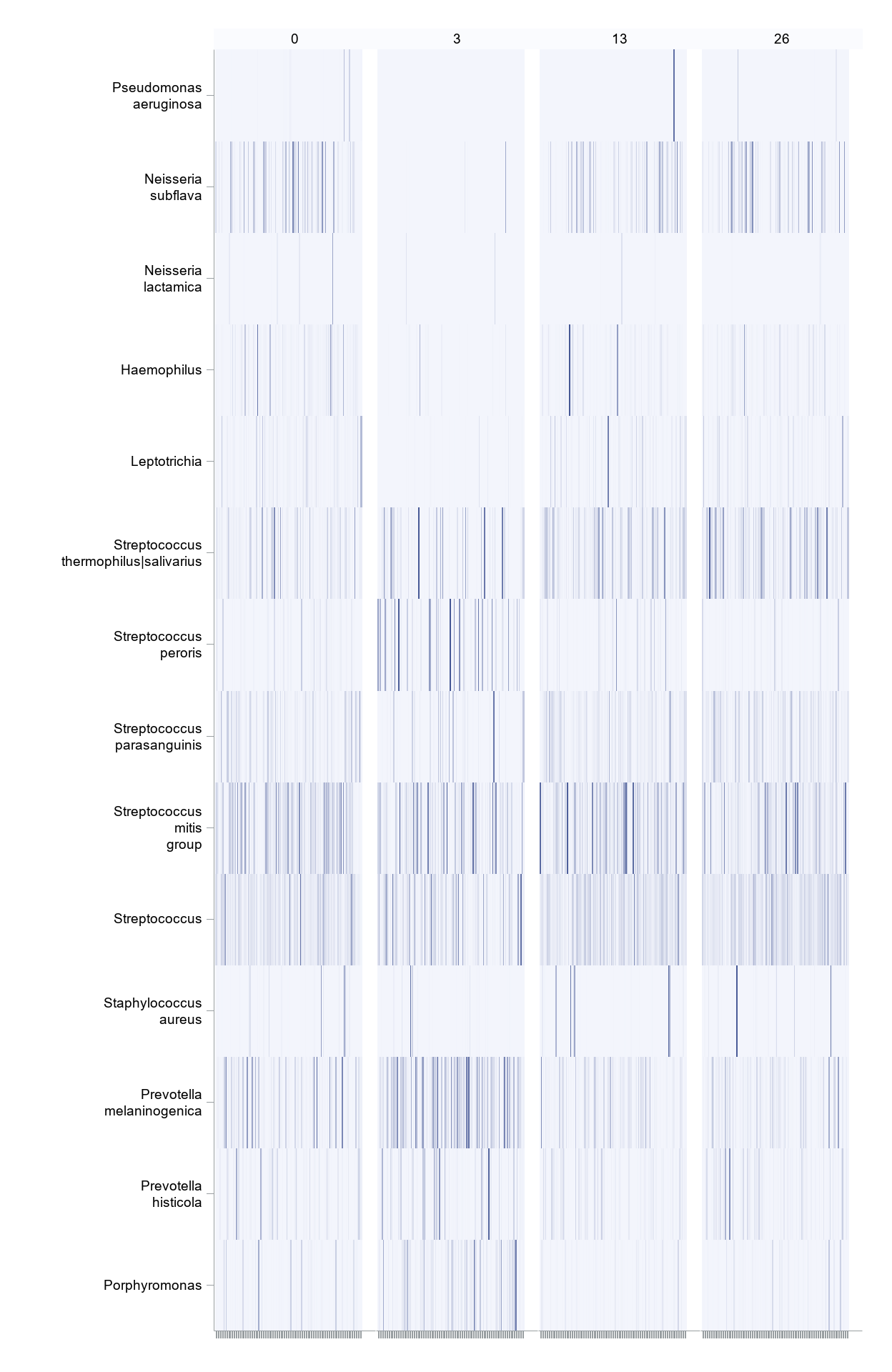


Figure E4. Bacterial communities over time. Heatmap with taxa presented in rows, samples in columns, the darker blue indicates higher RA.


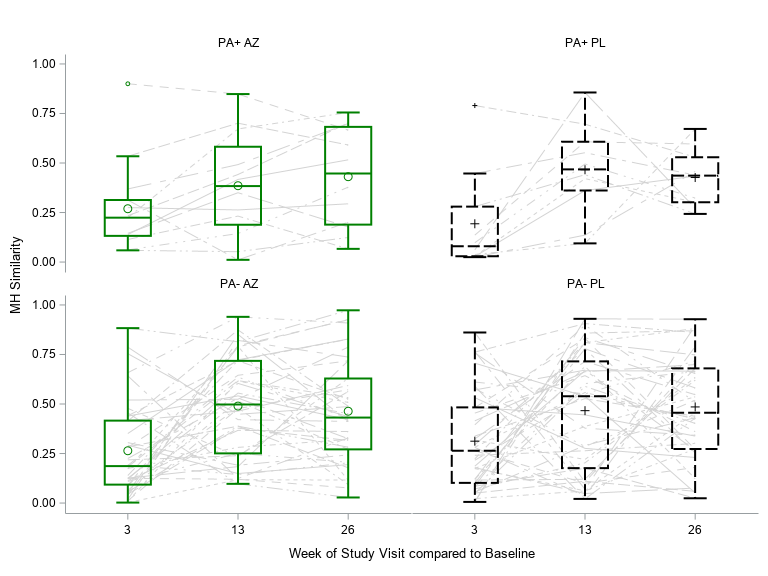


Figure E5. Distribution of MH by PA stratified cohorts comparing communities at each study visit to baseline.


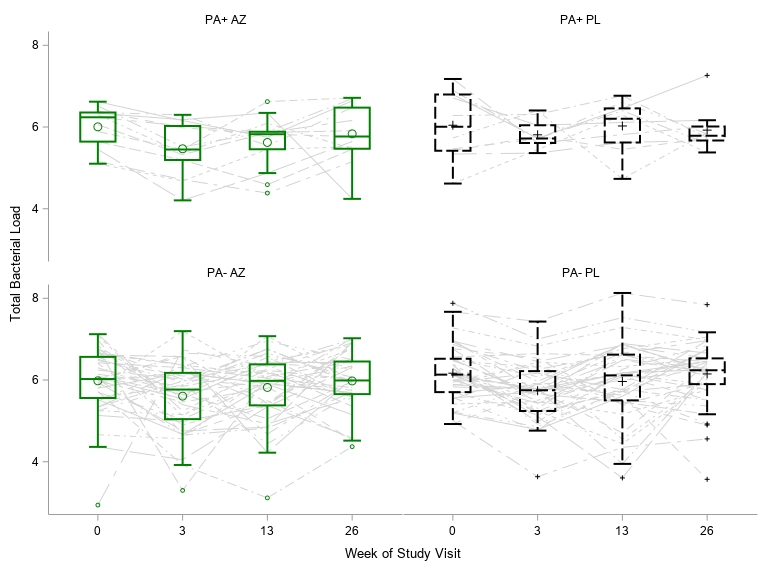

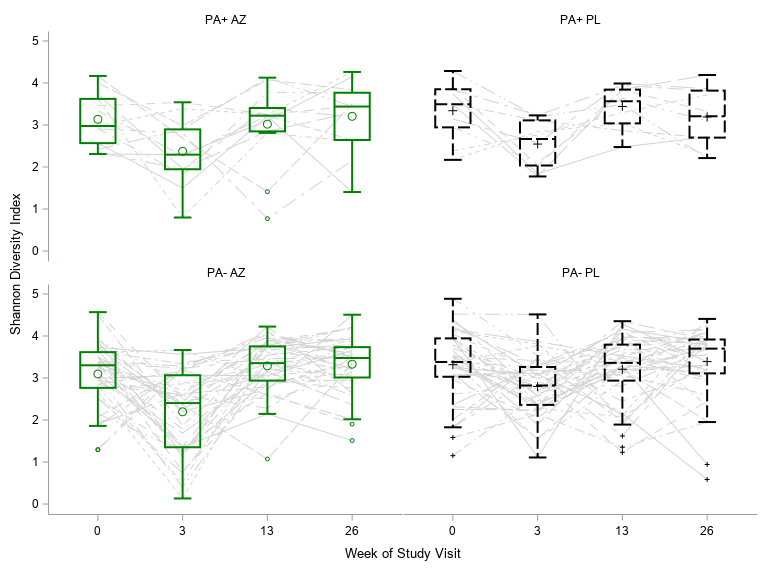
Figure E6. Distribution of change in TBL and SDI over time by PA stratified cohorts. TBL=total bacterial load; SDI = Shannon Diversity Index


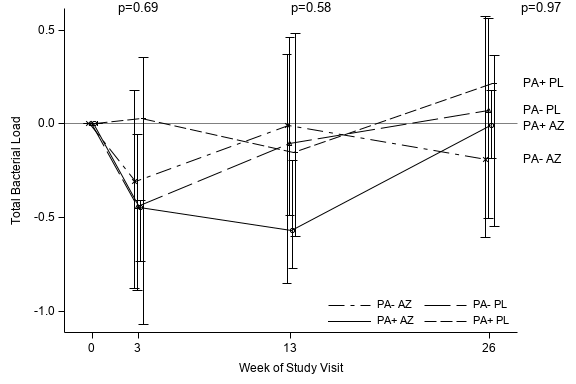

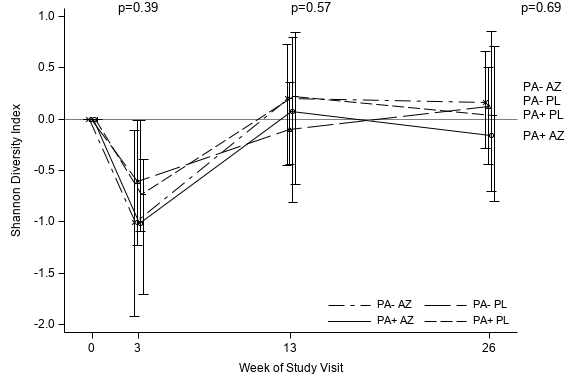

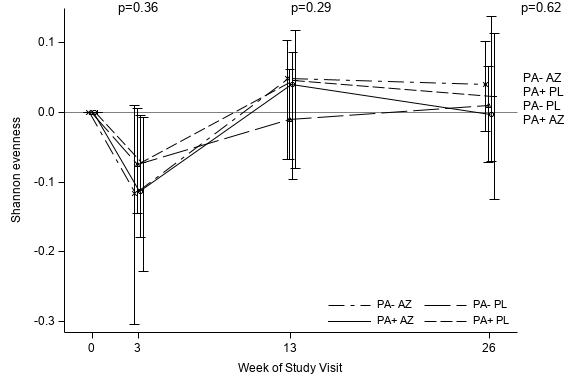

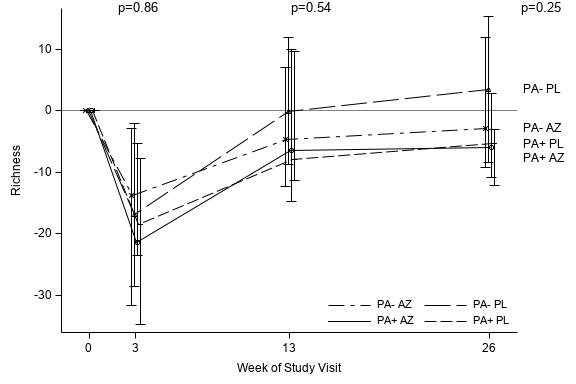

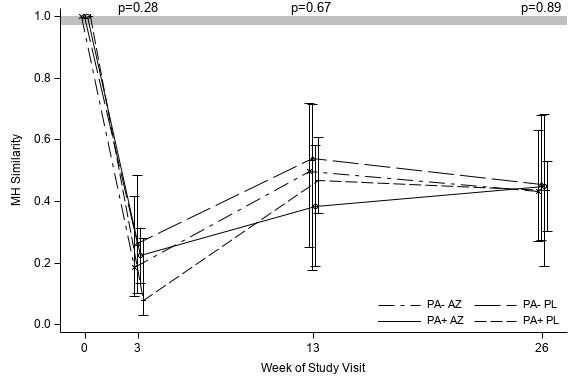

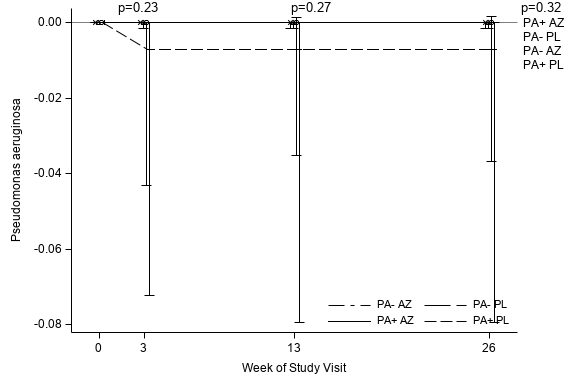

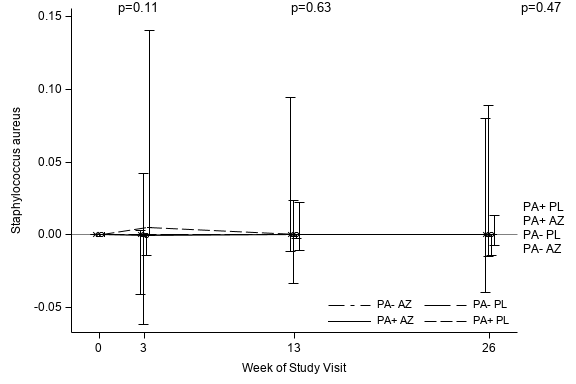

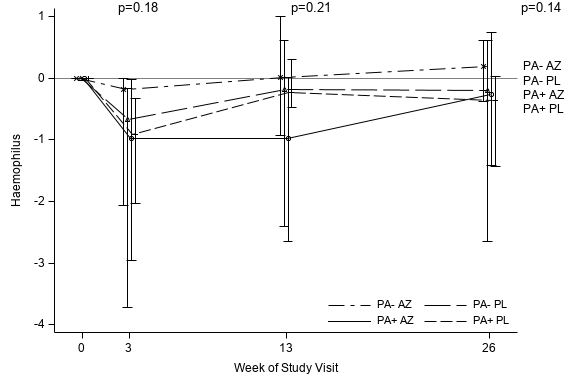

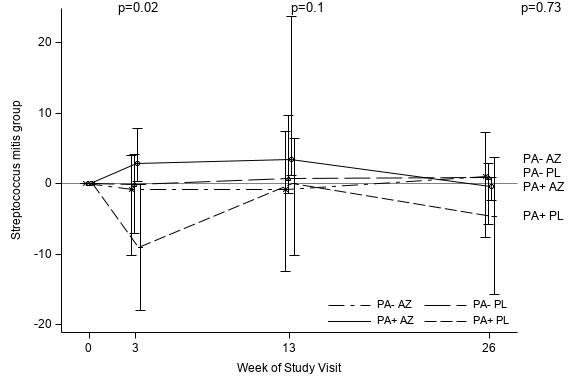

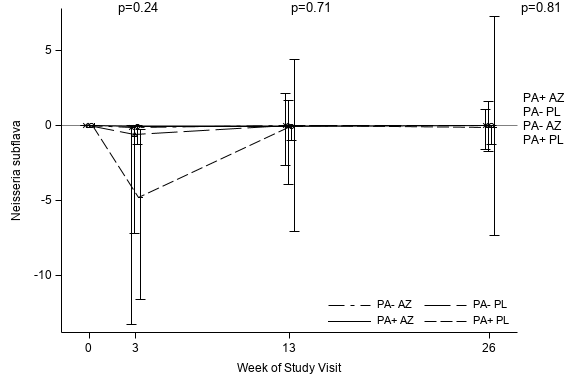

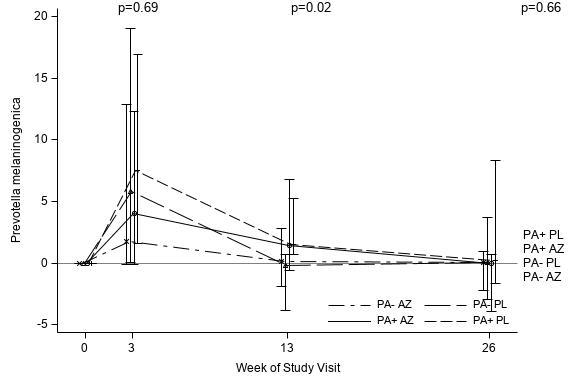

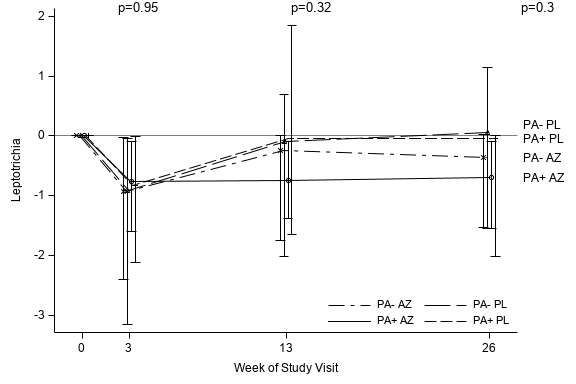


Figure E7. Change from baseline for microbial measures by PA stratified cohorts


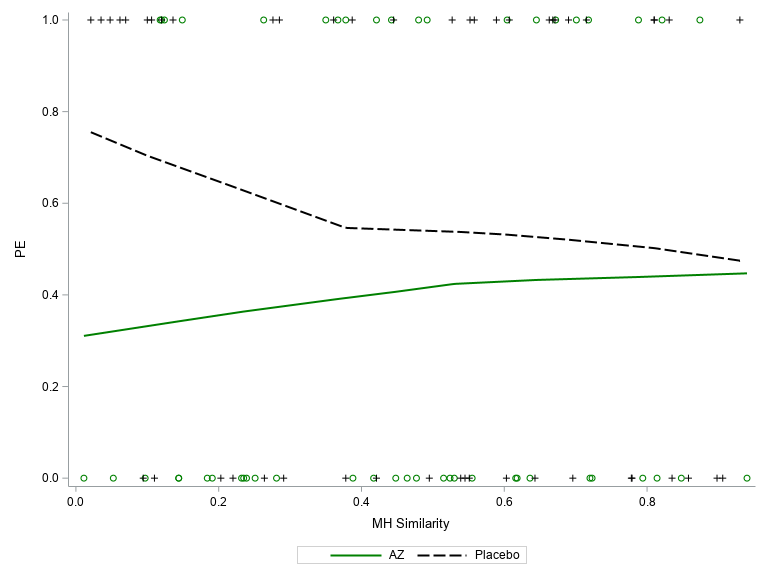

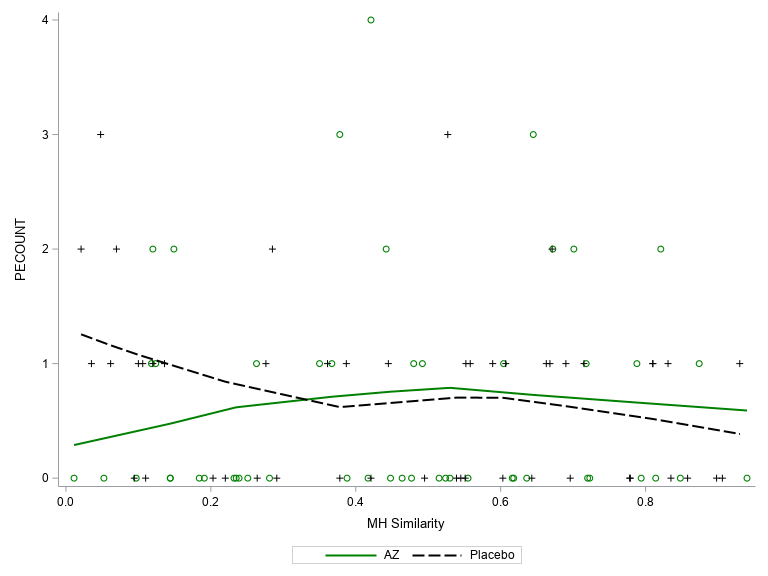
Figure E8. PEx outcomes (any PEx (top) 0=no, 1=yes; number of PEx (bottom)) plotted versus MH_0-13_ stratified by treatment.


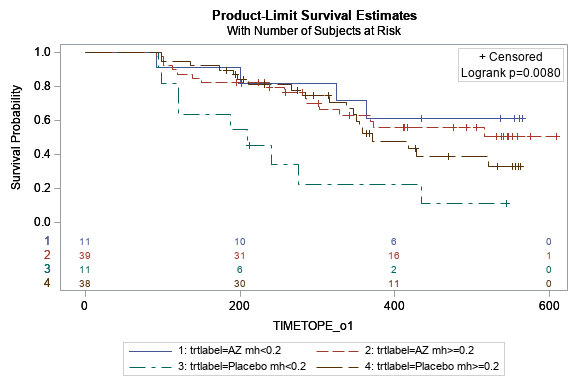


Figure E9. Time to PEx based on initial eradication treatment group stratified by community stability (MH < 0.2). bacterial community profiles for 22 individuals with large shifts in community composition between baseline and week 13 had differential response to treatment.


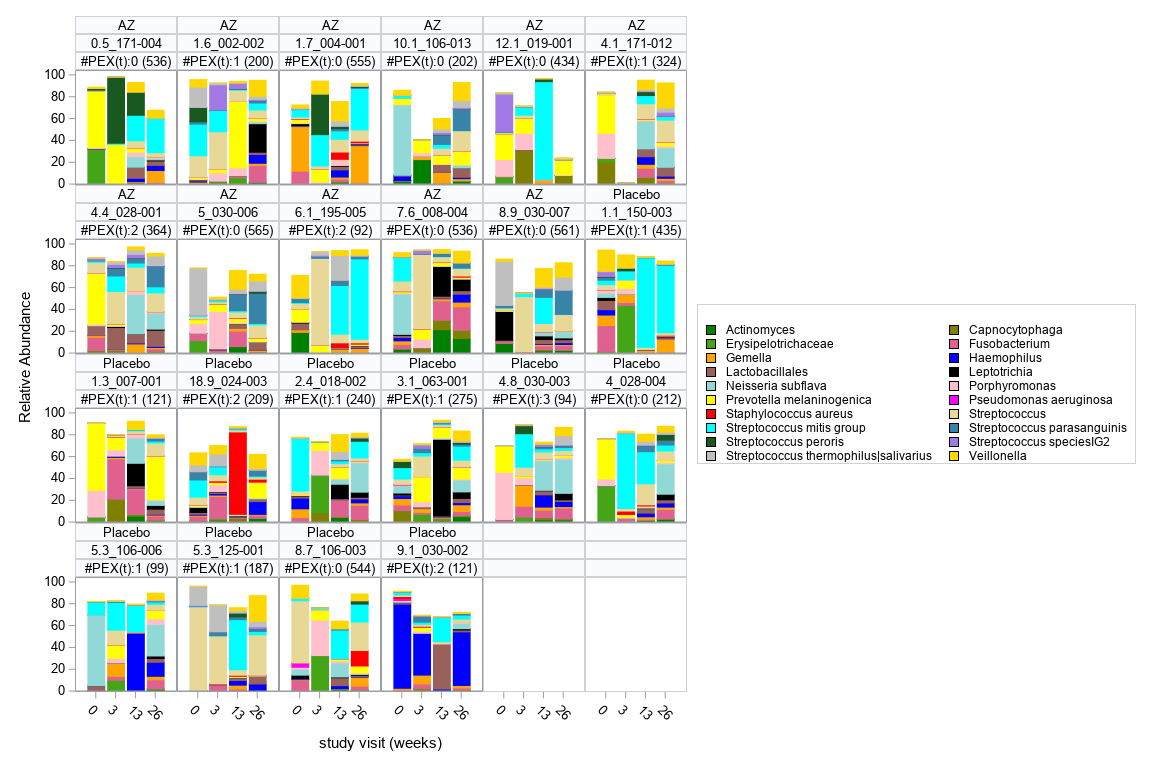


Figure E10. Microbial community composition for 22 subjects with low MH_0-13_. Each subject is displayed in a separate panels showings amples from each study visit. Labels on top of panels represent the randomized treatment group, age, de-identified id, number and days to 1^st^ PEx (or censoring time if 0 PEx).


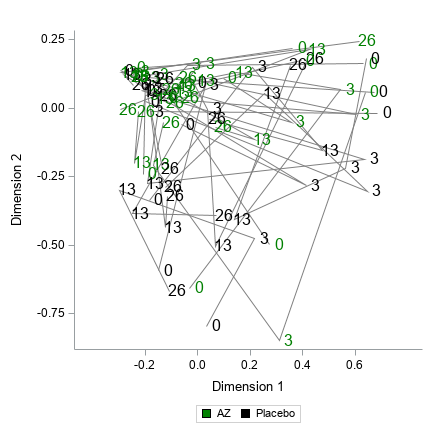

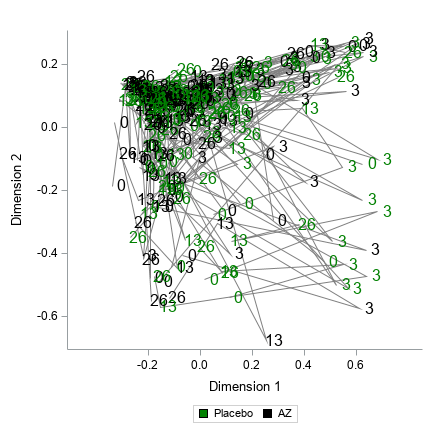


Figure E11. PCoA showing whether the 22 subjects with low MH_0-13_ are converging to a similar community (top), subjects with higher MH_0-13_ are plotted for comparison (bottom).

**List of 45 centers at which this research was performed.**

Alaska Locations

**[Anchorage, Alaska, United States, 99519-6604](https://clinicaltrials.gov/)**

[CFF Affiliate Program Providence Medical Center](https://clinicaltrials.gov/)

**Arizona Locations**

**[Tucson, Arizona, United States, 85724](https://clinicaltrials.gov/)**

[CFF Care Center Arizona Health Science Center](https://clinicaltrials.gov/)

**Arkansas Locations**

**[Little Rock, Arkansas, United States, 72202](https://clinicaltrials.gov/)**

[CFF Care Center & Pediatric Program Arkansas Children's Hospital](https://clinicaltrials.gov/)

**California Locations**

**[Los Angeles, California, United States, 90027](https://clinicaltrials.gov/)**

[Childrens Hospital Los Angeles](https://clinicaltrials.gov/)

**[Palo Alto, California, United States, 94304](https://clinicaltrials.gov/)**

[CFF Care Center & Pediatric Program Stanford University](https://clinicaltrials.gov/)

**Colorado Locations**

**[Aurora, Colorado, United States, 80045](https://clinicaltrials.gov/)**

[CFF Care Center & Pediatric Program Children's Hospital Colorado](https://clinicaltrials.gov/)

**Connecticut Locations**

**[New Haven, Connecticut, United States, 06520](https://clinicaltrials.gov/)**

[CFF Care Center & Pediatric Program Yale University](https://clinicaltrials.gov/)

**Florida Locations**

**[Jacksonville, Florida, United States, 32207](https://clinicaltrials.gov/)**

[CFF Care Center & Pediatric Program Nemours Children's Clinic - Jacksonville](https://clinicaltrials.gov/)

**[Saint Petersburg, Florida, United States, 33701](https://clinicaltrials.gov/)**

[CFF Care Center & Pediatric Program All Children's Hospital](https://clinicaltrials.gov/)

**Georgia Locations**

**[Atlanta, Georgia, United States, 30324](https://clinicaltrials.gov/)**

[CFF Care Center & Pediatric Program Emory University](https://clinicaltrials.gov/)

**[Atlanta, Georgia, United States, 30342](https://clinicaltrials.gov/)**

[CFF Affiliate Program Children's Healthcare of Atlanta](https://clinicaltrials.gov/)

**Idaho Locations**

**[Boise, Idaho, United States, 83712](https://clinicaltrials.gov/)**

[CFF Care Center St. Luke's CF Clinic](https://clinicaltrials.gov/)

**Illinois Locations**

**[Chicago, Illinois, United States, 60611-2605](https://clinicaltrials.gov/)**

[CFF Care Center & Pediatric Program Ann & Robert H. Lurie Children's Hospital of Chicago](https://clinicaltrials.gov/)

**Indiana Locations**

**[Indianapolis, Indiana, United States, 46202-5271](https://clinicaltrials.gov/)**

[CFF Care Center & Pediatric Program Riley Hospital for Children](https://clinicaltrials.gov/)

**Iowa Locations**

**[Iowa City, Iowa, United States, 52242](https://clinicaltrials.gov/)**

[CFF Care Center & Pediatric Program University of Iowa](https://clinicaltrials.gov/)

**Maine Locations**

**[Portland, Maine, United States, 04102](https://clinicaltrials.gov/)**

[CFF Care Center & Pediatric Program Maine Medical Center](https://clinicaltrials.gov/)

**Massachusetts Locations**

**[Boston, Massachusetts, United States, 02115](https://clinicaltrials.gov/)**

[CFF Care Center & Pediatric Program Children's Hospital Boston](https://clinicaltrials.gov/)

**Michigan Locations**

**[Ann Arbor, Michigan, United States, 48109-5212](https://clinicaltrials.gov/)**

[CFF Care Center & Pediatric Program University of Michigan](https://clinicaltrials.gov/)

**[Detroit, Michigan, United States, 48201](https://clinicaltrials.gov/)**

[CFF Care Center & Pediatric Program Children's Hospital of Michigan](https://clinicaltrials.gov/)

**Missouri Locations**

**[Kansas City, Missouri, United States, 64108](https://clinicaltrials.gov/)**

[CFF Care Center The Children's Mercy Hospital](https://clinicaltrials.gov/)

**[Saint Louis, Missouri, United States, 63104](https://clinicaltrials.gov/)**

[CFF Care Center & Pediatric Program Cardinal Glennon Children's Hospital/Saint Louis University](https://clinicaltrials.gov/)

**[Saint Louis, Missouri, United States, 63110](https://clinicaltrials.gov/)**

[CFF Care Center & Pediatric Program St. Louis Children's Hospital](https://clinicaltrials.gov/)

**Nebraska Locations**

**[Omaha, Nebraska, United States, 68198](https://clinicaltrials.gov/)**

[CFF Care Center & Pediatric Program University of Nebraska Medical Center](https://clinicaltrials.gov/)

**New Jersey Locations**

**[Long Branch, New Jersey, United States, 07740](https://clinicaltrials.gov/)**

[CFF Care Center & Pediatric Program Monmouth Medical Center](https://clinicaltrials.gov/)

**New York Locations**

**[New York, New York, United States, 10032](https://clinicaltrials.gov/)**

[CFF Care Center & Pediatric Program Columbia University](https://clinicaltrials.gov/)

**[Syracuse, New York, United States, 13210](https://clinicaltrials.gov/)**

[CFF Care Center & Pediatric Program SUNY Upstate Medical University](https://clinicaltrials.gov/)

**[Valhalla, New York, United States, 10595](https://clinicaltrials.gov/)**

[CFF Care Center New York Medical College](https://clinicaltrials.gov/)

**North Carolina Locations**

**[Chapel Hill, North Carolina, United States, 27599](https://clinicaltrials.gov/)**

[CFF Care Center & Pediatric Program University of North Carolina at Chapel Hill](https://clinicaltrials.gov/)

**Ohio Locations**

**[Akron, Ohio, United States, 44308](https://clinicaltrials.gov/)**

[CFF Care Center & Pediatric Program Akron Children's Hospital](https://clinicaltrials.gov/)

**[Cincinnati, Ohio, United States, 45229](https://clinicaltrials.gov/)**

[CFF Care Center & Pediatric Program Cincinnati Children's Hospital Medical Center](https://clinicaltrials.gov/)

**[Cleveland, Ohio, United States, 44106](https://clinicaltrials.gov/)**

[CFF Care Center & Pediatric Program Rainbow Babies and Children's Hospital](https://clinicaltrials.gov/)

**[Columbus, Ohio, United States, 43205](https://clinicaltrials.gov/)**

[CFF Care Center & Pediatric Program Nationwide Children's Hospital](https://clinicaltrials.gov/)

**[Dayton, Ohio, United States, 45404](https://clinicaltrials.gov/)**

[CFF Care Center & Pediatric Program The Children's Medical Center](https://clinicaltrials.gov/)

**Oregon Locations**

**[Portland, Oregon, United States, 97239](https://clinicaltrials.gov/)**

[CFF Care Center & Pediatric Program Oregon Health & Sciences University](https://clinicaltrials.gov/)

**Pennsylvania Locations**

**[Hershey, Pennsylvania, United States, 17033](https://clinicaltrials.gov/)**

[CFF Care Center & Pediatric Program Hershey Medical Center](https://clinicaltrials.gov/)

**[Pittsburgh, Pennsylvania, United States, 15224](https://clinicaltrials.gov/)**

[CFF Care Center & Pediatric Program Children's Hospital of Pittsburgh](https://clinicaltrials.gov/)

**South Dakota Locations**

**[Sioux Falls, South Dakota, United States, 57117](https://clinicaltrials.gov/)**

[CFF Care Center & Pediatric Program Sanford USD Medical Center](https://clinicaltrials.gov/)

**Tennessee Locations**

**[Memphis, Tennessee, United States, 38103](https://clinicaltrials.gov/)**

[CFF Care Center & Pediatric Program University of Tennessee](https://clinicaltrials.gov/)

**Texas Locations**

**[Austin, Texas, United States, 78723](https://clinicaltrials.gov/)**

[CFF Care Center & Pediatric Program Dell Children's Medical Center of Central Texas](https://clinicaltrials.gov/)

**Utah Locations**

**[Salt Lake City, Utah, United States, 84132](https://clinicaltrials.gov/)**

[University of Utah](https://clinicaltrials.gov/)

**Virginia Locations**

**[Norfolk, Virginia, United States, 23507](https://clinicaltrials.gov/)**

[CFF Care Center & Pediatric Program Children's Hospital of the King's Daughters](https://clinicaltrials.gov/)

**[Richmond, Virginia, United States, 23298](https://clinicaltrials.gov/)**

[CFF Care Center Medical College of Virginia](https://clinicaltrials.gov/)

**Washington Locations**

**[Seattle, Washington, United States, 98145](https://clinicaltrials.gov/)**

[CFF Care Center & Pediatric Program Seattle Children's Hospital](https://clinicaltrials.gov/)

**Wisconsin Locations**

**[Madison, Wisconsin, United States, 53792](https://clinicaltrials.gov/)**

[CFF Care Center & Pediatric Program University of Wisconsin](https://clinicaltrials.gov/)

**[Milwaukee, Wisconsin, United States, 53226](https://clinicaltrials.gov/)**

[CFF Care Center & Pediatric Program Children's Hospital of Wisconsin](https://clinicaltrials.gov/)

References

1. Borowitz D, Robinson KA, Rosenfeld M, Davis SD, Sabadosa KA, Spear SL, Michel SH, Parad RB, White TB, Farrell PM, Marshall BC, Accurso FJ. Cystic Fibrosis Foundation evidence-based guidelines for management of infants with cystic fibrosis. *J Pediatr* 2009; 155: S73-93.

2. Saiman L, Siegel J. Infection control recommendations for patients with cystic fibrosis: Microbiology, important pathogens, and infection control practices to prevent patient-to-patient transmission. *Am J Infect Control* 2003; 31: S1-62.

3. Nadkarni MA, Martin FE, Jacques NA, Hunter N. Determination of bacterial load by real-time pcr using a broad-range (universal) probe and primers set. *Microbiology* 2002; 148: 257-266.

4. Zemanick ET, Wagner BD, Sagel SD, Stevens MJ, Accurso FJ, Harris JK. Reliability of quantitative real-time pcr for bacterial detection in cystic fibrosis airway specimens. *PloS One* 2010; 5: e15101.

5. Langmead B, Salzberg SL. Fast gapped-read alignment with bowtie 2. *Nat Methods* 2012; 9: 357-359.

6. Illumina. Igenomes. 2017. Available from: <https://support.illumina.com/sequencing/sequencing_software/igenome.html>.

7. Hara N, Alkanani AK, Ir D, Robertson CE, Wagner BD, Frank DN, Zipris D. Prevention of virus-induced type 1 diabetes with antibiotic therapy. *Journal of Immunology* 2012; 189: 3805-3814.

8. Markle JG, Frank DN, Mortin-Toth S, Robertson CE, Feazel LM, Rolle-Kampczyk U, von Bergen M, McCoy KD, Macpherson AJ, Danska JS. Sex differences in the gut microbiome drive hormone-dependent regulation of autoimmunity. *Science* 2013; 339: 1084-1088.

9. Zemanick ET, Wagner BD, Robertson CE, Ahrens RC, Chmiel JF, Clancy JP, Gibson RL, Harris WT, Kurland G, Laguna TA, McColley SA, McCoy K, Retsch-Bogart G, Sobush KT, Zeitlin PL, Stevens MJ, Accurso FJ, Sagel SD, Harris JK. Airway microbiota across age and disease spectrum in cystic fibrosis. *Eur Respir J* 2017; 50.

10. Ewing B, Green P. Base-calling of automated sequencer traces using phred. Ii. Error probabilities. *Genome Research* 1998; 8: 186-194.

11. Ewing B, Hillier L, Wendl MC, Green P. Base-calling of automated sequencer traces using phred. I. Accuracy assessment. *Genome Research* 1998; 8: 175-185.

12. Edgar RC, Haas BJ, Clemente JC, Quince C, Knight R. Uchime improves sensitivity and speed of chimera detection. *Bioinformatics* 2011; 27: 2194-2200.

13. Schloss PD, Westcott SL. Assessing and improving methods used in operational taxonomic unit-based approaches for 16s rrna gene sequence analysis. *Appl Environ Microbiol* 2011; 77: 3219-3226.

14. Pruesse E, Peplies J, Glockner FO. Sina: Accurate high-throughput multiple sequence alignment of ribosomal rna genes. *Bioinformatics* 2012; 28: 1823-1829.

15. Quast C, Pruesse E, Yilmaz P, Gerken J, Schweer T, Yarza P, Peplies J, Glockner FO. The silva ribosomal rna gene database project: Improved data processing and web-based tools. *Nucleic Acids Research* 2013; 41: D590-596.

16. Robertson CE, Harris JK, Wagner BD, Granger D, Browne K, Tatem B, Feazel LM, Park K, Pace NR, Frank DN. Explicet: Graphical user interface software for the management, analysis and visualization of microbial ecology data and metadata. *Bioinformatics* 2013.
